# The role of kidney transplantation and phosphate binder use in vitamin K status

**DOI:** 10.1371/journal.pone.0203157

**Published:** 2018-08-30

**Authors:** Thijs T. Jansz, Aegida Neradova, Adriana J. van Ballegooijen, Marianne C. Verhaar, Marc G. Vervloet, Leon J. Schurgers, Brigit C. van Jaarsveld

**Affiliations:** 1 Department of Nephrology and Hypertension, University Medical Center Utrecht, Utrecht University, Utrecht, the Netherlands; 2 Department of Nephrology and Cardiovascular Sciences (ACS), VU University Medical Center, Amsterdam, the Netherlands; 3 Department of Epidemiology and Biostatistics, VU University Medical Center, Amsterdam, the Netherlands; 4 Amsterdam Public Health Institute, VU University Medical Center, Amsterdam, the Netherlands; 5 Department of Biochemistry, Cardiovascular Research Institute Maastricht, University Maastricht, Maastricht, the Netherlands; 6 Dianet Dialysis Centers, Utrecht, the Netherlands; Nagoya University, JAPAN

## Abstract

**Background:**

Cardiovascular disease is the leading cause of death in end-stage renal disease and is strongly associated with vascular calcification. Both kidney transplantation and phosphate binders may lower the risk of vascular calcification. Vascular calcification is actively inhibited by vitamin-K-dependent matrix γ-carboxyglutamic acid protein (MGP). Whether kidney transplantation or phosphate binders affect vitamin K status is unknown. Therefore, we studied the influence of kidney transplantation and phosphate binder use on vitamin K status.

**Methods:**

We measured plasma desphospho-uncarboxylated MGP (dp-ucMGP), a marker reflecting low vitamin K status, in a cross-sectional study of patients on hemodialysis *(n = 82)*, peritoneal dialysis *(n = 31)* or who recently received a kidney transplantation *(n = 36)*. By medication inventory, we assessed phosphate binder use. With linear regression, we assessed the influence of kidney transplantation and phosphate binder use on natural-log-transformed dp-ucMGP, adjusting for potential confounders.

**Results:**

Mean age of patients was 52±13 years; 102 (68%) were male. Dp-ucMGP levels were significantly lower in kidney transplant recipients (median 689 pmol/L) compared to patients on dialysis (median 1537 pmol/L, p<0.001). Eighty-nine patients on dialysis used phosphate binders. Using any phosphate binder was not associated with dp-ucMGP levels (median 1637 pmol/L, p = 0.09) compared to no phosphate binders (median 1142 pmol/L). Twenty-six patients used sevelamer monotherapy, which was associated with higher dp-ucMGP levels (median 1740 pmol/L, p = 0.04) after adjusting for age, sex and vitamin K antagonist use.

**Conclusions:**

Recent kidney transplantation is associated with lower dp-ucMGP levels suggesting improved vitamin K status after transplantation. Sevelamer monotherapy is associated with higher dp-ucMGP levels suggesting worsening of vitamin K status. Both findings warrant more attention to vitamin K status in patients on dialysis, as vitamin K is necessary for protection against vascular calcification.

## Introduction

Cardiovascular disease accounts for over 50% of deaths in end-stage renal disease (ESRD)[1], and is advanced by vascular calcification, often encountered in ESRD[2–4]. Vascular calcification is inhibited by matrix γ-carboxyglutamic acid protein (MGP)[5], which needs vitamin K for carboxylation to its active form. High levels of inactive MGP, desphospho-uncarboxylated MGP (dp-ucMGP), indicate vitamin K deficiency[6] and are associated with vascular calcification in chronic kidney disease[7, 8].

Kidney transplantation is the preferred treatment for ESRD, and rapidly normalizes serum phosphate[9]. Phosphate contributes importantly to vascular calcification[10]. However, kidney transplantation does not normalize cardiovascular disease risk, even though it prolongs life expectancy[11–13]. Interestingly, kidney transplant recipients are often vitamin K deficient[14], which is associated with increased mortality[15].

Patients on dialysis are routinely prescribed phosphate binders to lower serum phosphate levels. Despite their widespread use, definite proof for beneficial outcomes is lacking[16]. Remarkably, phosphate binder use did not improve and even worsened vascular calcification compared to placebo in a randomized trial in patients with chronic kidney disease, despite lowering of slightly elevated baseline phosphate levels[17]. Therefore, it is conceivable that phosphate binders exert adverse effects that are yet unknown[17].

Recent *in-vitro* studies indicate that various phosphate binders may not only bind phosphate, but also fat-soluble vitamins such as vitamin K[18–20]. Phosphate binders might thus worsen vitamin K status and hence promote vascular calcification.

It is unclear whether kidney transplantation improves low vitamin K status in patients on dialysis[21]. Moreover, it is unknown whether phosphate binder use affects vitamin K status in patients on dialysis. Therefore, we measured dp-ucMGP in a cohort of patients with ESRD, to assess whether kidney transplantation is associated with better vitamin K status, i.e. lower dp-ucMGP levels, as compared to hemodialysis and peritoneal dialysis. Finally, we investigated whether use of phosphate binders was associated with worse vitamin K status, i.e. higher dp-ucMGP levels in patients on dialysis.

## Materials and methods

### Study population

We analyzed a cross-sectional cohort from the ongoing NOCTx study (NCT00950573), a prospective cohort study that included prevalent patients on hemodialysis and peritoneal dialysis with a minimum dialysis vintage of 2 months, and patients who received a kidney transplant 2–3 months before inclusion. Patients were eligible when aged between 18 and 75 years, and were candidates for transplantation when on dialysis. All study participants gave written informed consent. NOCTx excluded patients with a life expectancy <3 months, pre-emptive transplantation, non-adherence to dialysis regimens, drug abuse, and pregnancy. NOCTx has been approved by the Medical Ethics Committee of the University Medical Centre Utrecht and is conducted according to the Declaration of Helsinki. None of the transplant donors were from a vulnerable population and all donors or next of kin provided written informed consent that was freely given.

Between December 2009 and February 2016, NOCTx included 181 patients who were referred for study participation to the University Medical Centre of Utrecht, the Netherlands, by 8 Dutch dialysis centers. Patients were treated according to guidelines by the attending nephrologists. For kidney transplant recipients, standard immunosuppressant regimens consisted of a calcineurin inhibitor (tacrolimus), mycophenolate mofetil, and prednisone in tapering doses.

Blood samples were collected in 4.5 mL potassium-ethylenediaminetetraacetic acid vacutainers (on a non-dialysis day in case of hemodialysis), immediately centrifuged and stored in aliquots at -80°C without thawing. For the present analyses, we excluded patients whose blood samples were not available *(n = 28*) or with missing data for medication prescriptions *(n = 4*), leaving a final sample of 149 patients, 113 of which were on dialysis.

### Dp-ucMGP measurements

We determined vitamin K status by measuring dp-ucMGP. Dp-ucMGP is a sensitive marker of vitamin K status, as opposed to circulating vitamin K_1_ and K_2_, which may fluctuate substantially as a result of dietary intake[21] and degrade when exposed to light[22]. Plasma dp-ucMGP levels were determined using the commercially available IVD CE-marked chemiluminescent InaKtif MGP assay on the IDS-iSYS system (IDS, Boldon, United Kingdom). Patient sample and internal calibrators were incubated with magnetic particles coated with murine monoclonal antibodies dpMGP, acridinium-labelled murine monoclonal antibodies ucMGP, and an assay buffer. The magnetic particles were captured using a magnet and washed to remove any unbound analyte. Trigger reagents were added; the resulting light emitted by the acridinium label was directly proportional to the level of dp-ucMGP in the sample. The within-run and total variations of this assay were 0.8–6.2% and 3.0–8.2%, respectively. The assay measuring range was between 300 and 12,000 pmol/L and was linear up to 11,651 pmol/L[8]. All assays were performed in a single run by the laboratory of Coagulation Profile, department of Biochemistry, Maastricht, the Netherlands.

### Phosphate binder use

Study personnel recorded phosphate binder use with lists of prescribed medication at time of blood sample collection. Similarly, vitamin K antagonist (VKA) and vitamin D analog use were recorded. We categorized patients according to phosphate binder use: no binders or any binder, subcategorized as exclusively non-calcium containing binders or exclusively calcium containing binders. Additionally, we subcategorized patients according to monotherapy with a single binder (sevelamer hydrochloride or carbonate, lanthanum carbonate, calcium carbonate, calcium acetate/magnesium carbonate, or calcium acetate).

### Other study variables

At time of sampling, study personnel recorded demographic and clinical parameters (pre-dialysis blood pressure and post-dialysis weight averaged from routine measurements during 3 hemodialysis sessions or 2 outpatient visits). Total calcium, albumin, phosphate, parathyroid hormone, C-reactive protein and total cholesterol were routinely measured at local treatment facilities (pre-dialysis for patients on hemodialysis). Smoking status and height were self-reported. Study personnel evaluated residual urine production with a 24h-urine collection. We assessed history of kidney disease, current dialysis schedule and presence of comorbidities by chart review. We defined diabetes mellitus as the necessity for oral diabetes medication or insulin therapy, and cardiovascular disease as any history of angina, myocardial infarction, percutaneous coronary intervention, coronary artery bypass grafting, aortic aneurysm ≥5 cm, stroke, intermittent claudication, peripheral artery angioplasty or bypass grafting. We defined dialysis vintage as the time since the first day of dialysis, minus the time with a functioning kidney transplant. For kidney transplant recipients, we estimated glomerular filtration rate with the Chronic Kidney Disease Epidemiology Collaboration (CKD-EPI) 2009 equation.

### Statistical analyses

We present results as mean (± standard deviation) for normally distributed variables, as median (interquartile range, IQR) for non-normally distributed variables, or as number (percentage) for categorical data. We tabulated baseline characteristics and medication use by renal replacement therapy. We compared dialysis vintage and dp-ucMGP between groups with Mann-Whitney-U tests.

The distribution of dp-ucMGP levels was right-skewed and we therefore natural-log-transformed dp-ucMGP levels. With boxplots and linear regression we examined the associations of both renal replacement therapy and phosphate binder use with log-transformed dp-ucMGP. We compared log-transformed dp-ucMGP between kidney transplantation and dialysis (hemodialysis and peritoneal dialysis jointly), and between hemodialysis and peritoneal dialysis. We compared log-transformed dp-ucMGP between phosphate binder categories in patients on dialysis only, as none of the kidney transplant recipients used phosphate binders. We compared patients using any phosphate binder, exclusively non-calcium containing phosphate binders, exclusively calcium containing phosphate binders and sevelamer monotherapy to non-phosphate binder users. We adjusted stepwise for potential confounders age (years), sex (male/female), VKA use (yes/no), intensive hemodialysis regimens (≥18h/week or <18h/week) and residual urine production (≥100mL/24h or absent). Additionally, we analyzed non-VKA users only. In the final models of both renal replacement therapy and phosphate binder use, we adjusted for age (years), sex (male/female) and VKA use (yes/no).

We reported regression coefficients with 95% confidence intervals (95% CI). Regression coefficients should be interpreted multiplicatively, i.e. as a ratio, after exponentiation. We considered P-values of ≤ 0.05 (two-tailed) statistically significant and performed all analyses with R 3.3.3 (R Foundation Statistical Computing).

## Results

### Study population

The mean age of the study population *(n = 149)* was 52 ±13 years, 102 patients (68%) were male. Dialysis vintage (including historical dialysis vintage of kidney transplant recipients) was 23 months (IQR 11–49 months), 21 patients (14%) had diabetes mellitus and 16 patients (11%) used VKAs (acenocoumarol or phenprocoumon; 4 kidney transplant recipients and 12 patients on hemodialysis). Eighty-two patients were treated with hemodialysis, 31 with peritoneal dialysis and 36 were kidney transplant recipients (Table 1).

**Table 1:** Baseline characteristics of the 149 patients with end-stage renal disease stratified by renal replacement therapy.

		Hemo- dialysis (n = 82)	Peritoneal dialysis (n = 31)	Kidney transplantation (n = 36)
***Demographics and medical history***				
**Age (y)**	53.3 ±12.2		49.8 ±14.2	50.4 ±15.1
**Male (%)**	53 (65%)		21 (68%)	28 (78%)
**BMI (kg/m**^**2**^**)**	26.1 ±5.0		24.2 ±3.3	24.9 ±3.3
**Systolic blood pressure (mmHg)**	140 ±19		134 ±13	128 ±13
**Diastolic blood pressure (mmHg)**	77 ±11		84 ±12	77 ±7
**Diabetes mellitus (%)**	17 (21%)		1 (3%)	3 (8%)
**Prior cardiovascular disease (%)**	19 (23%)		6 (19%)	5 (14%)
**Active smoker (%)**	13 (16%)		5 (16%)	4 (11%)
***History of kidney disease***				
***Dialysis vintage (months)***	28 (13–59)		12 (6–22)	31 (12–55)
**Cause of ESRD (%)**				
• **Cystic kidney disease**	9 (11%)		3 (10%)	12 (33%)
• **Interstitial nephritis**	3 (4%)		1 (3%)	1 (3%)
• **Glomerulonephritis**	27 (33%)		7 (23%)	6 (17%)
• **Vascular disease**	15 (18%)		7 (23%)	7 (19%)
• **Diabetic nephropathy**	9 (11%)		0 (0%)	3 (8%)
• **Other**	13 (16%)		7 (23%)	3 (8%)
• **Unknown**	6 (7%)		6 (19%)	4 (11%)
**Dialysis therapy and kidney function**				
**Dialysis therapy**				
• **Weekly HD sessions**	3.5 ±0.9		-	-
• **Weekly HD hours**	16.9 ±11.1		-	-
• **Daily PD dwells**	-		4.4 ±0.6	-
• **Daily PD volume (L)**	-		9.3 ±2.1	-
**Kidney function**				
• **Residual urine production ≥100mL/24h (%)**	38 (46%)		21 (68%)	-
• **eGFR (mL/min)**	-		-	53 ±20
***Laboratory parameters***				
**Calcium (mmol/L)**	2.3 ±0.2		2.3 ±0.1	2.4 ±0.1
**Albumin (g/L)**	41.5 ±3.2		38.7 ±3.1	40.3 ±3.2
**Phosphate (mmol/L)**	1.7 ±0.4		1.6 ±0.4	0.8 ±0.3
**Parathyroid hormone (pmol/L)**	20 (11–43)		21 (14–42)	13 (10–27)
**C-reactive protein (mg/L)**	3.0 (2.0–5.0)		1.5 (1.0–6.8)	3.0 (1.5–5.0)
**Total cholesterol (mmol/L)**	4.4 ±1.1		5.1 ±1.6	4.8 ±1.2
**dp-ucMGP (pmol/L)**	1605 (993–2390)		1195 (921–1807)	689 (489–1078)

Data are presented as mean ±standard deviation, median (interquartile range) or number (percentage). Abbreviations: BMI: body mass index; ESRD: end-stage renal disease; eGFR: estimated glomerular filtration rate, calculated with the Chronic Kidney Disease-Epidemiology Collaboration equation 2009; dp-ucMGP: desphospho-uncarboxylated matrix Gla-protein.

The mean age of patients not included in the evaluation sample *(n = 32* without samples or medication lists) was 52±13 years, 19 (59%) were male, dialysis vintage was 32 months (IQR 15–69 months, P = 0.15 versus study population) and 5 patients (16%) had diabetes mellitus. Nineteen of these patients were treated with hemodialysis, 7 with peritoneal dialysis and 6 were kidney transplant recipients.

### Phosphate binder use

Twenty-four of the 113 patients on dialysis (21%) did not use phosphate binders, while 89 (79%) used any phosphate binder. Fifty-three of these patients used exclusively non-calcium containing phosphate binders, whereas 10 used exclusively calcium containing phosphate binders (Table 2). Thirty-eight of the patients on dialysis (34%) used a single phosphate binder, 45 patients (40%) two types of phosphate binders, and 6 patients (5%) three types of phosphate binders. None of the kidney transplant recipient used phosphate binders, apart from 500 mg calcium carbonate once daily, commonly prescribed as supplement post-transplantation *(n = 22)*. We therefore excluded kidney transplant recipients from the phosphate binder analyses.

**Table 2:** Medication prescriptions in 149 patients with end-stage renal disease, stratified by renal replacement therapy.

	
	Hemo- dialysis (n = 82)	Peritoneal dialysis (n = 31)	Kidney transplantation (n = 36)
**Vitamin K antagonists (%)**	12 (15%)	0 (0%)	4 (11%)
**Vitamin D analogs (%)**	57 (70%)	25 (81%)	4 (11%)
**Any phosphate binder (%)**	60 (73%)	29 (94%)	0 (0%)
• **Exclusively non-calcium containing phosphate binders (%)**	36 (44%)	17 (55%)	0 (0%)
○ **Sevelamer monotherapy (%)**	20 (24%)	6 (19%)	0 (0%)
• **Exclusively calcium containing phosphate binders (%)**	6 (7%)	4 (13%)	0 (0%)

Data are presented as numbers (percentage). Vitamin K antagonists prescribed were acenocoumarol and phenprocoumon, and vitamin D analogs prescribed were alfacalcidol.

Overall, sevelamer was the most frequently prescribed phosphate binder (73 patients, 65%), followed by lanthanum carbonate (37 patients, 33%), calcium carbonate (29 patients, 26%), calcium acetate/magnesium carbonate (4 patients, 4%) and calcium acetate (3 patients, 3%). Sevelamer was prescribed as monotherapy in 26 patients (23%). Monotherapy with lanthanum carbonate, calcium carbonate, calcium acetate/magnesium carbonate and calcium acetate occurred too infrequently (in 2, 8, 1 and 1 patients, respectively) and was therefore not studied separately.

### Dp-ucMGP levels

Median plasma levels of dp-ucMGP in all 149 patients with ESRD were 1302 (IQR 739–1838) pmol/L. Dp-ucMGP levels were highest in VKA users (median 4718, IQR 3028–6672 versus 1146, IQR 704–1716 pmol/L in non-users, P<0.001). Dp-ucMGP levels were right-skewed and therefore natural-log-transformed for regression analyses.

### Associations between kidney transplantation and dp-ucMGP

**Fig 1:**
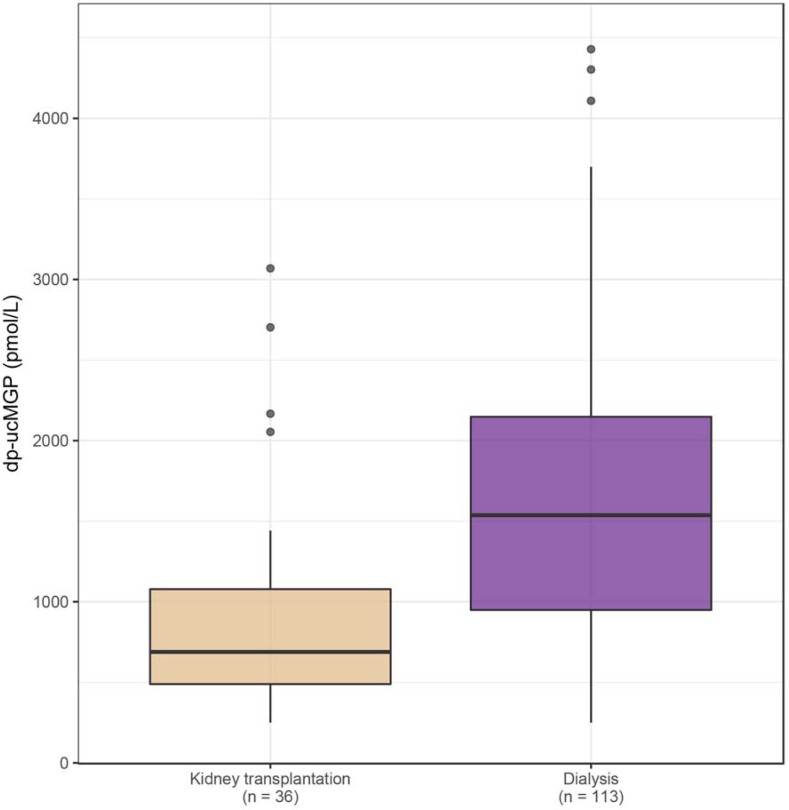
Dp-ucMGP levels stratified by renal replacement therapy, as boxplots. Kidney transplant recipients *(n = 36)*: sand-color boxplot, and both patients on hemo- and peritoneal dialysis *(n = 113)*: purple boxplot. Median values of dp-ucMGP: 689 and 1537 pmol/L respectively.

In kidney transplant recipients, median dp-ucMGP levels were 689 (IQR 489–1078) pmol/L. In patients on hemodialysis, median dp-ucMGP levels were 1605 (IQR 993–2390) pmol/L, and in patients on peritoneal dialysis 1195 (IQR 921–1807) pmol/L (Fig 1). In crude regression analyses, dp-ucMGP levels were lower in kidney transplant recipients as compared to patients treated with hemo- and peritoneal dialysis (regression coefficient -0.64, 95% confidence interval [CI] -0.91; -0.36). This association remained numerically similar when adjusted for age, sex and VKA use (regression coefficient -0.61, 95% CI -0.84; -0.37). Hemodialysis was associated with higher dp-ucMGP levels compared to peritoneal dialysis in crude regression analyses (regression coefficient 0.26, 95% CI -0.05; 0.56), but not when adjusted for age, sex and VKA use (regression coefficient 0.05, 95% CI -0.31; 0.21). The above associations remained numerically similar when analyzed in non-VKA users only (S1 Table).

### Associations between phosphate binder use and dp-ucMGP

**Fig 2:**
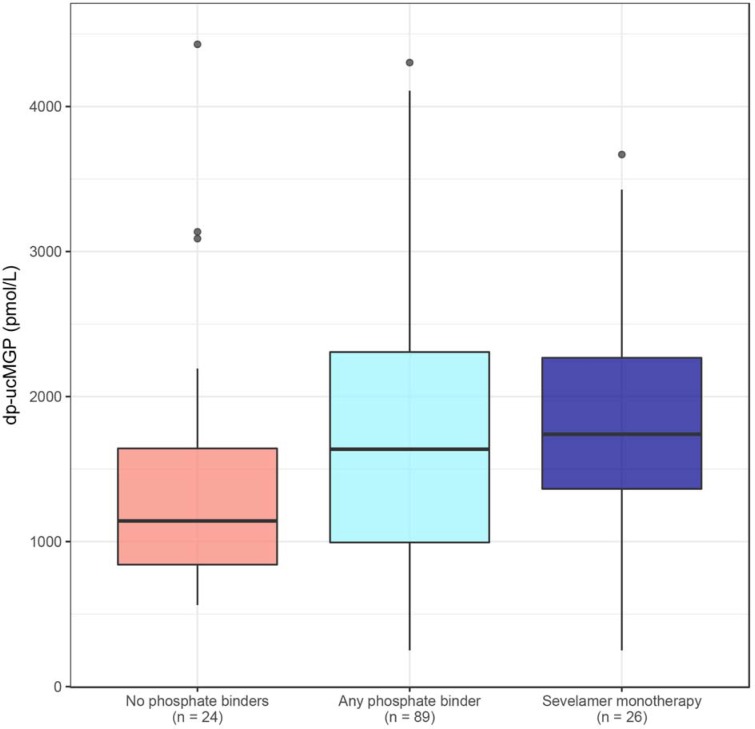
Dp-ucMGP levels stratified by phosphate binder use in patients on dialysis, as boxplots. Patients using no phosphate binders *(n = 24)*: pink boxplot, patients using any phosphate binder *(n = 89)*: light blue boxplot, and a subgroup of patients using sevelamer monotherapy *(n = 26)*: dark blue boxplot. Median values of dp-ucMGP: 1142; 1637; and 1740 pmol/L, respectively.

In patients on dialysis without phosphate binders *(n* = *24*), median dp-ucMGP levels were 1142 (IQR 841–1642) pmol/L, while in those on any phosphate binder *(n = 89)* median dp-ucMGP levels were 1637 (IQR 994–2307) pmol/L. This difference was non-significant, and remained numerically similar when adjusted for age, sex and VKA use (Table 3). In patients on dialysis on exclusively non-calcium containing phosphate binders *(n = 53)* and exclusively calcium containing phosphate binders *(n = 10)*, median dp-ucMGP levels were 1615 (IQR 1068–2061) and 2330 (IQR 1014–3476) pmol/L, respectively. Compared to patients on dialysis without phosphate binders, these dp-ucMGP levels were not significantly different, and the differences remained numerically similar in the adjusted model. In patients on dialysis on sevelamer monotherapy *(n = 26)*, median dp-ucMGP levels were 1740 (IQR 1363–2267) pmol/L. These dp-ucMGP levels were significantly higher compared to patients on dialysis without phosphate binders in the adjusted model (Fig 2). The above associations remained numerically similar when analyzed in non-VKA users only (S2 Table).

**Table 3:** Regression coefficients of linear regression analysis of phosphate binder prescription and log-transformed dp-ucMGP levels in 113 patients with end-stage renal disease on dialysis.

	N	Crude regression coefficient 95% CI	Adjusted regression coefficient* 95% CI
**No phosphate binders**	24	0.0 *(reference)*	0.0 *(reference)*
**Any phosphate binder**	89	0.26 (-0.08; 0.60)	0.25 (-0.04; 0.53)
• **Exclusively non-calcium containing phosphate binder**	53	0.23 (-0.10; 0.56)	0.28 (-0.02; 0.59)
○ **Sevelamer monotherapy**	26	0.34 (-0.04; 0.72)	0.35 (0.02; 0.68)
• **Exclusively calcium containing phosphate binder**	10	0.42 (-0.12; 0.95)	0.28 (-0.20; 0.76)

Plasma dp-ucMGP was skewed to the right and therefore log-transformed. Hence, regression coefficients should be interpreted as a ratio after exponentiation. CI: confidence interval

*Adjusted for age (years), sex (male/female) and vitamin K antagonist use (yes/no).

## Discussion

Our study shows that kidney transplant recipients have substantially lower dp-ucMGP levels compared to patients on any form of dialysis, indicating better vitamin K status after restoration of kidney function. Phosphate binder use in general is not associated with dp-ucMGP levels. However, sevelamer monotherapy is associated with significantly higher dp-ucMGP levels compared to no phosphate binders, suggesting a negative effect of sevelamer on vitamin K status.

To our knowledge, vitamin K status and dp-ucMGP levels have only been studied in kidney transplant recipients or patients on dialysis in isolation. Previous studies have examined stable kidney transplant recipients (median 6 years after transplantation) with a similar age to our population (mean and median age 51[15] and 56 years[14]), and found similar dp-ucMGP levels to our measurements 2–3 months after transplantation[14, 15]. A previous study of healthy individuals (mean age 53 years) has reported slightly lower dp-ucMGP levels (447 ±188 pmol/L) as compared to kidney transplant recipients in our study (median 689 pmol/L)[23]. In our study, kidney transplant recipients had about twice as low dp-ucMGP levels compared to patients on dialysis (median 689 versus 1537 pmol/L). Likely, dp-ucMGP levels in kidney transplant recipients have been higher before transplantation, because they had all been on dialysis. Altogether, this suggests that vitamin K status improves rapidly after kidney transplantation.

There are several possible explanations for the lower dp-ucMGP levels in kidney transplant recipients. Uremia impairs carboxylation of MGP by reducing γ-carboxylase[24]; thus, relief of uremia by kidney transplantation might restore carboxylation capacity of MGP as reflected by lower dp-ucMGP levels. Furthermore, dp-ucMGP levels could be lower because of improved vitamin K intake after kidney transplantation; vitamin K intake is poor in patients on dialysis, partly due to the dietary restrictions[21]. Nonetheless, vitamin K deficiency was still common after transplantation, reflected by dp-ucMGP >500 pmol/L[23] in 20 out of 32 kidney transplant recipients that did not use VKAs.

We found lower dp-ucMGP levels in patients on hemodialysis compared to previous literature. These include cross-sectional studies that examined older patients on hemodialysis (median age 74[8] and 65 years[21]), as well as a randomized trial on vitamin K_2_ supplementation (mean age 64 years[25]). Interestingly, in our study dp-ucMGP levels were higher among patients on hemodialysis than on peritoneal dialysis, which could be explained by patients on hemodialysis using VKAs more frequently. Frequent VKA use may predispose patients on hemodialysis to vitamin K deficiency. Possibly, this difference will be attenuated when patients on dialysis will use direct oral anticoagulants instead of VKAs in the future.

Recent *in vitro* studies have investigated the impact of phosphate binders on vitamin K, demonstrating that most classes of binders can bind fat-soluble vitamins including vitamin K[18, 19]. This implies that phosphate binders may aggravate vitamin K deficiency, hampering activation of MGP and limiting protection against vascular calcification. Currently, little is known about the impact of phosphate binders on vitamin K status in humans. Counterintuitively, an observational study in patients on hemodialysis has reported lower total ucMGP levels with calcium carbonate use[26]. However, total ucMGP reflects vitamin K status poorly[23], unlike dp-ucMGP, which was not measured. To our knowledge, no human study has investigated the impact of phosphate binders on vitamin K status, as measured by dp-ucMGP.

Our study shows an association between sevelamer monotherapy and higher dp-ucMGP levels after adjusting for age, sex and VKA use, but remarkably no association between phosphate binder use in general and dp-ucMGP levels, although we did observe a trend towards higher dp-ucMGP levels. This discrepancy could be explained by confounding by better dietary intake, including vitamin K intake, by patients using multiple phosphate binders. These patients had higher phosphate levels (1.74±0.30 mmol/L) compared to patients using a single phosphate binder (1.58±0.39 mmol/L), which might suggest better dietary intake. On the other hand, the supposed vitamin K-lowering effect of phosphate binders could have been masked by medication non-adherence in patients using multiple phosphate binders.

Our findings may be clinically relevant: the better vitamin K status after kidney transplantation may in part explain the lower risk of cardiovascular disease after transplantation[13], given that high dp-ucMGP is associated with vascular calcification and all-cause mortality in chronic kidney disease[27]. This therefore warrants more attention to vitamin K status in patients on dialysis by improving dietary vitamin K intake, or even by vitamin K supplementation. Furthermore, it is a safety signal that sevelamer may negatively affect vitamin K status; the same may apply to other phosphate binders when tested in larger studies. Possibly, this supposed effect of phosphate binder use caused the remarkable progression of vascular calcification seen in the study by Block et al[17]. This placebo-controlled randomized trial found worsening of vascular calcification in patients allocated to phosphate binders compared to placebo, despite lowering of serum phosphate[17]. When confirmed, this justifies even more attention to vitamin K status of patients on dialysis, especially of those on phosphate binders.

Our study has some limitations. First, there may have been indication bias for no phosphate binder use. Using no phosphate binders may have been related to malnutrition, and hence a lower production of various proteins, including MGP and thereby dp-ucMGP. Conversely, malnutrition could also have increased dp-ucMGP levels by poor vitamin K intake. Importantly, both albumin levels and BMI did not suggest malnutrition in non-phosphate binder users (albumin 42.4 ±3.2 g/L and BMI 26.2 ±4.4 kg/m^2^ versus 40.3 ±3.4 g/L and 25.4 ±4.8 in kg/m^2^ in phosphate binder users). A second limitation is that the aim of the main study was about dialysis modality and vascular calcification, and hence dietary intake and medication adherence were not measured. On the one hand, dosages of phosphate binders were therefore not taken into account. On the other hand, medication non-adherence could have protected patients from iatrogenic vitamin K deficiency by phosphate binders, masking the supposed adverse effect. Third, monotherapy with phosphate binders other than sevelamer occurred too infrequently to be analyzed separately. Finally, we did not measure vitamin D status, which might also be affected by phosphate binder use[28], or other MGP species, such as total ucMGP and total MGP. Nevertheless, dp-ucMGP remains the most accurate marker of vitamin K status of all MGP species[23, 29].

Our study also has several strengths. First, dp-ucMGP levels were measured in a single run with a validated, standardized technique. Second, patients in our study originated from 8 large dialysis centers, representing an urban as well as a rural population. We therefore believe our results can be generalized to other ESRD populations. Third, all patients on dialysis were transplantation candidates, while all kidney transplant recipient had been transplanted after a period on dialysis. Thus, patients on dialysis and kidney transplant recipients were comparable.

In conclusion, recent kidney transplantation is associated with lower dp-ucMGP levels, indicating rapid improvement of vitamin K status following restoration of kidney function. In patients on dialysis, sevelamer monotherapy is associated with higher dp-ucMGP levels compared to no phosphate binders, suggesting worsening of vitamin K status by this phosphate binder. This calls for attention to vitamin K status in patients on dialysis, as vitamin K is necessary for protection against vascular calcification.

## Supporting information

S1 Dataset Source data on which the results of this study are based. (CSV)

S1 Table Regression coefficients of linear regression analysis of renal replacement therapy and log-transformed dp-ucMGP levels in 149 patients with end-stage renal disease, stratified by vitamin K antagonist use. (PDF)

S2 Table Regression coefficients of linear regression analysis of phosphate binder use and log-transformed dp-ucMGP levels in 113 patients on dialysis, stratified by vitamin K antagonist use. (PDF)
