# Supplementary material for: The role of kidney transplantation and phosphate binder use in vitamin K status
Source: PLoS One. 2018 Aug 30;13(8):e0203157. doi: 10.1371/journal.pone.0203157 (PMC6117040; doi:10.1371/journal.pone.0203157)
Supplement: S1 Table — (PDF) [file pone.0203157.s002.pdf]

	All patients		Non-vitamin K antagonist users		Vitamin K antagonist users	
	N	Crude regression coefficient	N	Crude regression coefficient	N	Crude regression coefficient
**Hemo- and peritoneal dialysis**	113	0.0 *(reference)*	101	0.0 *(reference)*	12	0.0 *(reference)*
**Kidney transplantation**	36	-0.64 (-0.91; -0.36)	32	-0.64 (-0.89; -0.39)	4	-0.66 (-1.30; -0.02)
	**All patients**		**Non-vitamin K antagonist users**		**Vitamin K antagonist users**	
	**N**	**Crude regression coefficient**	**N**	**Crude regression coefficient**	**N**	**Crude regression coefficient**
**Peritoneal dialysis**	31	0.0 *(reference)*	31	0.0 *(reference)*	0	*n/a*
**Hemodialysis**	82	-0.26 (-0.57; 0.05)	70	-0.07 (-0.35; 0.21)	12	*n/a*
