# Supplementary material for: The role of kidney transplantation and phosphate binder use in vitamin K status
Source: PLoS One. 2018 Aug 30;13(8):e0203157. doi: 10.1371/journal.pone.0203157 (PMC6117040; doi:10.1371/journal.pone.0203157)
Supplement: S2 Table — (PDF) [file pone.0203157.s003.pdf]

	All patients		Non-vitamin K antagonist users		Vitamin K antagonist users	
	**N**	**Crude regression coefficient**	**N**	**Crude regression coefficient**	**N**	**Crude regression coefficient**
**No phosphate binders**	24	0.0 *(reference)*	22	0.0 *(reference)*	2	0.0 *(reference)*
**Any phosphate binder**	89	0.26 (-0.08; 0.60)	79	0.21 (-0.10; 0.53)	10	0.32 (-0.37; 1.01)
• **Exclusively non-calcium containing phosphate binder**	53	0.23 (-0.10; 0.56)	50	0.27 (-0.05; 0.60)	3	0.07 (-0.57; 0.70)
○ **Sevelamer monotherapy**	26	0.34 (-0.04; 0.72)	23	0.34 (-0.03; 0.70)	3	0.07 (-0.57; 0.70)
• **Exclusively calcium containing phosphate binder**	10	0.42 (-0.12; 0.95)	8	0.29 (-0.21; 0.79)	2	0.21 (-2.92; 3.34)
